# DFinder: a novel end-to-end graph embedding-based method to identify drug–food interactions

**DOI:** 10.1093/bioinformatics/btac837

**Published:** 2022-12-29

**Authors:** Tao Wang, Jinjin Yang, Yifu Xiao, Jingru Wang, Yuxian Wang, Xi Zeng, Yongtian Wang, Jiajie Peng

**Affiliations:** School of Computer Science, Northwestern Polytechnical University, Xi’an 710072, China; Key Laboratory of Big Data Storage and Management, Northwestern Polytechnical University, Ministry of Industry and Information Technology, Xi’an 710072, China; School of Computer Science, Northwestern Polytechnical University, Xi’an 710072, China; Key Laboratory of Big Data Storage and Management, Northwestern Polytechnical University, Ministry of Industry and Information Technology, Xi’an 710072, China; School of Computer Science, Northwestern Polytechnical University, Xi’an 710072, China; Key Laboratory of Big Data Storage and Management, Northwestern Polytechnical University, Ministry of Industry and Information Technology, Xi’an 710072, China; School of Computer Science, Northwestern Polytechnical University, Xi’an 710072, China; Key Laboratory of Big Data Storage and Management, Northwestern Polytechnical University, Ministry of Industry and Information Technology, Xi’an 710072, China; School of Computer Science, Northwestern Polytechnical University, Xi’an 710072, China; Key Laboratory of Big Data Storage and Management, Northwestern Polytechnical University, Ministry of Industry and Information Technology, Xi’an 710072, China; School of Computer Science, Northwestern Polytechnical University, Xi’an 710072, China; Key Laboratory of Big Data Storage and Management, Northwestern Polytechnical University, Ministry of Industry and Information Technology, Xi’an 710072, China; School of Computer Science, Northwestern Polytechnical University, Xi’an 710072, China; Key Laboratory of Big Data Storage and Management, Northwestern Polytechnical University, Ministry of Industry and Information Technology, Xi’an 710072, China; School of Computer Science, Northwestern Polytechnical University, Xi’an 710072, China; Key Laboratory of Big Data Storage and Management, Northwestern Polytechnical University, Ministry of Industry and Information Technology, Xi’an 710072, China

## Abstract

**Motivation:**

Drug–food interactions (DFIs) occur when some constituents of food affect the bioaccessibility or efficacy of the drug by involving in drug pharmacodynamic and/or pharmacokinetic processes. Many computational methods have achieved remarkable results in link prediction tasks between biological entities, which show the potential of computational methods in discovering novel DFIs. However, there are few computational approaches that pay attention to DFI identification. This is mainly due to the lack of DFI data. In addition, food is generally made up of a variety of chemical substances. The complexity of food makes it difficult to generate accurate feature representations for food. Therefore, it is urgent to develop effective computational approaches for learning the food feature representation and predicting DFIs.

**Results:**

In this article, we first collect DFI data from DrugBank and PubMed, respectively, to construct two datasets, named DrugBank-DFI and PubMed-DFI. Based on these two datasets, two DFI networks are constructed. Then, we propose a novel end-to-end graph embedding-based method named DFinder to identify DFIs. DFinder combines node attribute features and topological structure features to learn the representations of drugs and food constituents. In topology space, we adopt a simplified graph convolution network-based method to learn the topological structure features. In feature space, we use a deep neural network to extract attribute features from the original node attributes. The evaluation results indicate that DFinder performs better than other baseline methods.

**Availability and implementation:**

The source code is available at https://github.com/23AIBox/23AIBox-DFinder.

**Supplementary information:**

Supplementary data are available at *Bioinformatics* online.

## 1 Introduction

The efficacy of drugs will be affected by many factors, such as drug dosage, drug delivery route and concomitant drugs. Food, which is often taken with drugs, may also have a significant impact on the efficacy of drugs (Jensen *et al.*, 2015). Several studies (Bushra *et al.*, 2011; Schmidt and Dalhoff, 2002) have demonstrated that food can increase or decrease the activity of drugs or even cause adverse drug events (ADEs), which can be considered as drug–food interactions (DFIs).

In DFIs, food can affect the efficacy of drugs in three ways (Telessy, 2018): incompatibilities, pharmacokinetics (PK) and pharmacodynamics (PD). Incompatibility denotes the circumstance when the drug molecule and component of food bind together and produce an insoluble compound, which may hinder the bioaccessibility of the drug. The classical example is that tetracyclines cannot be absorbed because of the calcium content of dairy products (Neuvonen, 1976). The PK interactions mean that food components can enhance or hinder the movement of drug molecules in the body during the Absorption, Transport, Metabolism and Excretion phases. For example, grapefruit juice can inhibit the metabolism of cyclosporine in a short time after administration because of the inhibition of the cytochrome P450 enzyme (Hollander *et al.*, 1995). PD interactions are caused by specific pharmacological interactions between drugs and specific food components (Koziolek *et al.*, 2019). One of the examples is warfarin antagonizes the action of vitamin-K (originated from food, such as spinach) (Lurie *et al.*, 2010). Therefore, the understanding and study of DFIs can reduce ADEs and improve the absorption of nutrients and drugs. Unfortunately, the known DFIs are just the tip of the iceberg. There are still many DFIs to be identified.

Our current knowledge about DFIs mainly comes from clinical research and biomedical literature (Bushra *et al.*, 2011; Chen *et al.*, 2018; Otles and Senturk, 2014). However, these studies only involve a small number of drugs (anti-diabetic drugs, anti-tubercular drugs, anti-hypertensive drugs etc.) and limited food (grapefruit, mango, etc.). And most of them focus on revealing how food affects the efficacy of drugs from a biomedical perspective, which is time-consuming and expensive. Hence, computational approaches are needed to identify potential DFIs, rather than just obtaining DFIs from the literature. In drug–drug interactions (DDIs) and drug–target interactions identification tasks, many computational methods (Feng *et al.*, 2020; Peng *et al.*, 2021; Ryu *et al.*, 2018; You *et al.*, 2019) have been proposed and successfully used. These methods have made remarkable advances in solving these problems. However, to the best of our knowledge, there are not many computational methods designed for discovering DFIs. There are two main reasons. The first reason is the lack of systematic collection of DFI data. It is challenging to collect a large amount of DFIs data. The lack of gold standard data is a big obstacle for data-driven computational methods. Second, it is difficult to obtain a good feature representation of food; food is generally made up of a variety of chemical substances. Most descriptions of food so far are general and vague. The complexity of food makes it difficult to generate accurate food features. By now, only a few efforts have been made to identify DFIs. Ryu *et al.* (2018) propose DeepDDI, which first uses principal component analysis to reduce the dimension of the drug features and then feeds the learned features into a deep neural network (DNN) classifier. As an extended application, DeepDDI is applied to identify the interactions between drugs and food constituents. Although DeepDDI can be used to identify DFIs, it is mainly designed for identifying different types of DDIs. Ni *et al.* (2017) identify DFIs based on the overlap of proteins associated with drugs and food compounds. However, this method ignores several types of information, such as the attributes of drugs and food and the known interactions between drugs and food.

As a data structure, graphs (a.k.a. networks) contain a set of entities and relationships between these entities, which makes them very suitable for modeling biomedical systems (Yi *et al.*, 2022). In recent years, predicting interactions between biomedical entities is usually modeled as a link prediction problem on a graph (Wen *et al.*, 2018; Zitnik *et al.*, 2018). Similarly, the identification of DFIs can also be modeled as a link prediction task. In detail, DFIs can be represented by a network where nodes represent drugs or food and edges represent the relationships between food and drugs. For graph analysis, many graph embedding (a.k.a. graph representation learning) methods have been proposed. The goal of these methods is to learn a low-dimensional representation of each node while maximally preserving the structural information of graphs. The learned representation can be used as features for different downstream graph analysis tasks, such as link prediction (Peng *et al.*, 2019), node classification (Wang *et al.*, 2021), community detection (Wang *et al.*, 2020) and clustering (Yi *et al.*, 2022). Compared with traditional methods, which put much effort into feature engineering to develop biological features, graph embedding methods can learn better node representations automatically. Yue *et al.* (2020) group graph embedding methods into three categories: matrix factorization-based, random walk-based and neural network-based methods. MF-based methods aim to factorize a data matrix into lower-dimensional matrices while still keeping the manifold structure and topological properties hidden in the original data matrix (Ahmed *et al.*, 2013; Belkin and Niyogi, 2003; Cao *et al.*, 2015). Random walk-based methods try to learn node representations by generating node sequences through random walks in graphs, which generally have two steps. First, random walk is applied to generate node sequences in a graph. Then, the word2vec model (Mikolov *et al.*, 2013) is adopted to learn embeddings based on the generated sequences of nodes. In this way, the structural proximity of the graph can be preserved. Many neural network-based methods have been proposed for graph embedding, such as multilayer perceptron (Tang *et al.*, 2015), auto-encoder (Kipf and Welling, 2016b; Wang *et al.*, 2016), generative adversarial network (Wang *et al.*, 2019) and graph convolutional network (GCN) (Kipf and Welling, 2016a). Different methods adopt different neural architectures and use different kinds of graph information as input. Inspired by the success of the graph embedding methods in the biomedical link prediction tasks (Peng *et al.*, 2021; Sun *et al.*, 2020a; Zhao *et al.*, 2021), we propose a method called DFinder to identify the interactions between drugs and food. In addition, DFinder is designed in an end-to-end fashion for better optimizing all the parameters. To summarize, our contributions are 3-fold:

- To solve the lack of DFI resources, we construct two DFI datasets named DrugBank-DFI and PubMed-DFI. For DrugBank-DFI, we collect DFI-related information directly from the DrugBank database and then parse this information to generate the DrugBank-DFI dataset. For PubMed-DFI, we adopt the co-occurrence-based text mining method to obtain the PubMed-DFI dataset from PubMed.
- We propose a novel graph representation learning method named DFinder to learn better representations for drugs and food. DFinder combines node features and topological structures to learn the final representations of drugs and food constituents based on the DFI networks.
- The evaluation results show that DFinder outperforms the existing approaches for DFI prediction task.

## 2 Materials and methods

We propose a novel end-to-end graph embedding-based method named DFinder to identify DFIs based on the DFI network. Our work mainly contains two parts: DFI network construction and computational framework for DFI prediction. In the first part, we generate the DrugBank-DFI dataset from the DrugBank database and use the co-occurrence-based text mining method to obtain the PubMed-DFI dataset from PubMed. Then, we construct two DFI networks based on these two datasets, respectively. In the second part, we propose a graph embedding method to learn the low-dimensional representations of drugs and food constituents. We calculate interaction scores between drugs and food components using the inner product method based on low-dimensional representations and optimize our model by Bayesian Personalized Ranking (BPR) loss.

### 2.1 The construction of DFI networks

In this section, we introduce how to collect DFI data and construct DFI networks.

#### 2.1.1 DFI network based on DrugBank

We first collect DFI data from DrugBank (v 5.1.7) database, which contains specific information for each drug (i.e. drug interactions, pharmacology, chemical structures, targets, metabolism etc.) (Wishart *et al.*, 2006, 2008). We download the XML formatted DrugBank dataset, which stores sporadic information on DFIs. By parsing the XML document, we obtain about 3000 sentences describing information on DFI. We manually extract DFIs from these sentences. Among the 3000 sentences, some describe the influence of the time of taking medicine. For example, ‘Take on an empty stomach.’ and ‘Take at least 1 hour before eating a meal’. In addition, there are some sentences that do not show clearly the effects of specific foods on drugs, such as ‘Take with food. This increases the bioavailability of darolutamide.’ Therefore, we manually extract DFIs from sentences by screening out the mutex relationship, no-effect relationship and other unclear relationships between food constituent and drug.

The extracted food involved in DFIs can be divided into two types: food (e.g. avocado, figs and cheese) and food constituents (e.g. caffeine, vitamin c and zinc). It is hard to have a unified representation of these two types of food data. Therefore, we use the top 20 food constituents with high content in the food to represent the food. This is also consistent with the fact that the interaction between drugs and food is actually the interaction between drugs and food components (Ni *et al.*, 2017; Ryu *et al.*, 2018). We use the FooDB database (v 1.0) to obtain constituents of food. FooDB contains numerous records on foods, compounds, contents of food components and the mapping of food and its components. In addition, in the process of data acquisition, we only retain ‘small molecule’ types of drugs and food components. The final DrugBank-DFI dataset contains 1784 interactions, representing 143 drugs, and 213 food components. The density of the DrugBank-DFI bipartite network is 0.059, which is comparable with other DFI networks (Supplementary Table S1).

#### 2.1.2 DFI network based on PubMed

**Fig. 1.**
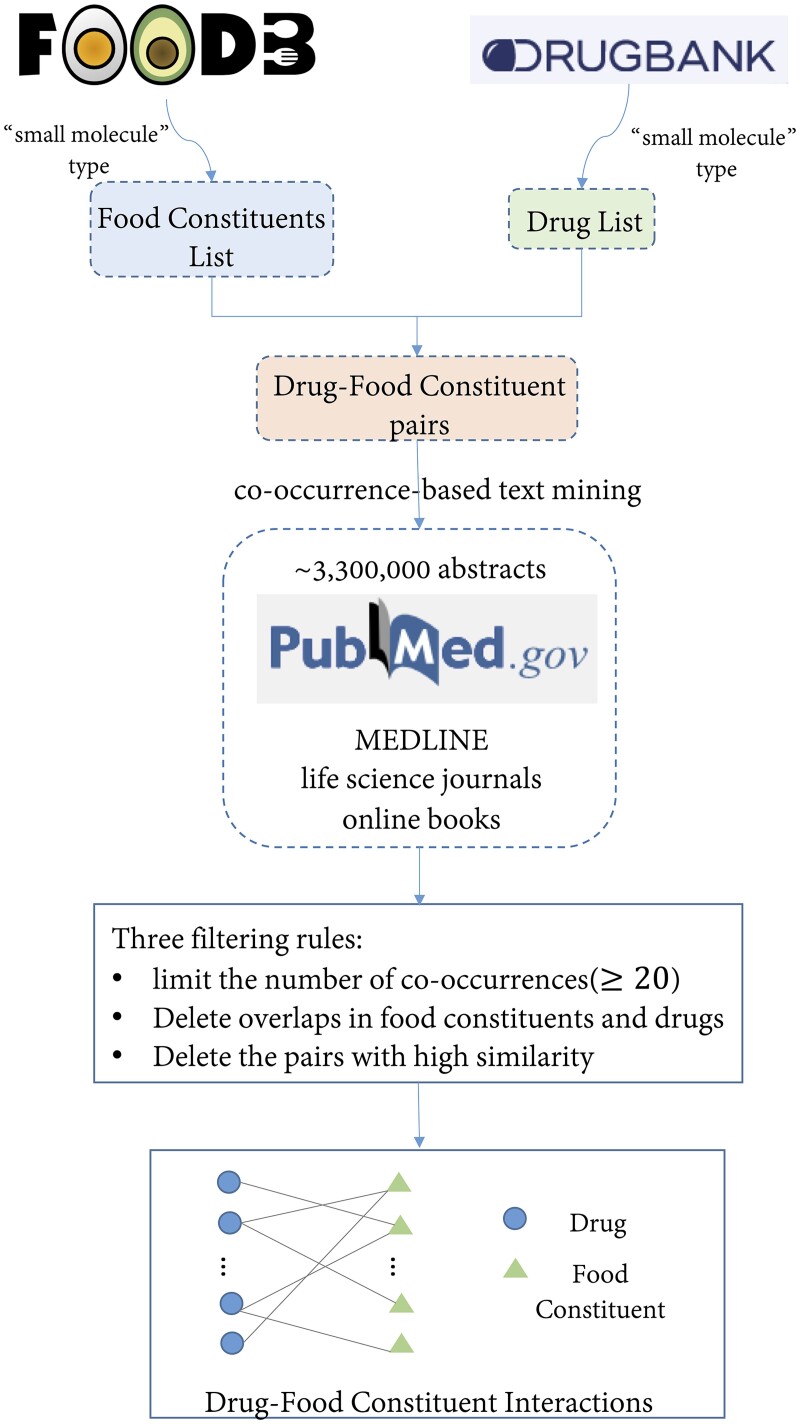
The flowchart of co-occurrence-based text mining method. Firstly, we obtain a candidate drug list from DrugBank and a candidate food constituent list from FooDB, respectively. Secondly, we combine all the drugs and food constituents in pairs. Thirdly, we input each drug–food constituent pair into PubMed to get the number of co-occurrence in abstracts of publications. Next, in order to improve the quality of data, we set up three rules to further filter the found drug–food constituent pairs. Finally, we use the filtered drug–food constituent pairs to construct a DFI network

Although some DFIs have been recorded in the DrugBank, more valuable DFIs are still buried in the biomedical literature. Therefore, we try to extract DFIs from the literature. Co-occurrence of biological entities in the literature is a simple, comprehensive and popular technique for identifying entity associations. The technique is based on the assumption that if a biological entity appears in the same document as another biological entity, the two entities should have a high probability of being biologically related (Fleuren and Alkema, 2015; Shanfeng *et al.*, 2005). This hypothesis was already validated in several existing studies (Junge and Jensen, 2020; Pletscher-Frankild *et al.*, 2015; Szklarczyk *et al.*, 2019). Therefore, we adopt a co-occurrence-based text mining method to obtain DFIs from the literature. The workflow of text mining is shown in Figure 1.

To aid this purpose, we use the standard PubMed Advanced search API (ncbi.nlm.nih.gov/books/NBK3827/#pubmedhelp.Advanced_Search) for text mining. In detail, we used the standard ‘ESearch’ utility (eutils.ncbi.nlm.nih.gov/entrez/eutils/esearch.fcgi) to query the occurrence of drug or food constituent, and we could output all of the paper IDs whose abstracts contain both the two entities using in-house scripts. As of October 2021, we searched about 3.3 million abstracts from PubMed. Similar to DrugBank-DFI, we only focus on ‘small molecule’ types of drugs and food components when mining DFIs from PubMed. Firstly, we obtain all small molecule drugs from the DrugBank dataset to form a list of candidate drugs. Then, we generate all food components in FooDB and screen out the small molecule components to form a candidate food component list. Secondly, we combined all the drugs and food components in pairs and then identify drug–food constituent pairs with potential interactions based on their co-occurrence in abstracts of publications in PubMed. Next, we set up three rules to further filter the identified drug–food constituent pairs for improving the quality of the dataset. The three rules are as follows:

- **Rule 1:** The number of co-occurrences should be larger than a given threshold. We only keep drug–food constituent pairs that co-occurred more than 20 times in PubMed.
- **Rule 2:** Since constituents of food and drugs may be the same, we removed the pairs including the same constituents.
- **Rule 3:** In some cases, the constituents of food and drugs are mentioned in the same article because they are similar. To avoid this problem, we calculated the similarity of the names of each pair and deleted the pairs with high similarity.

After applying these three rules, we obtain the PubMed-DFI dataset, which contains 15 890 drug–food constituent interactions, representing 779 drugs and 818 food constituents. The density of the PubMed-DFI bipartite graph is 0.025. We compare the detailed graph statistics of the two DFI datasets with other DFI and DDI networks and find that the sizes and densities of our DFI networks are reasonable (Supplementary Table S1).

To be noted, the PubMed-DFI is a co-occurrence-based DFI network, which might be affected by the not real interactions. But it is still valuable in developing and evaluating the robustness of our DFinder method since the co-occurrence-based FDIs largely expand the size of the drug–food bipartite network. Furthermore, the PubMed-DFI dataset may benefit further DFI validations by domain experts in the future. More about the co-occurrence-based DFI construction is discussed in the Supplementary Material.

### 2.2 Computational framework for DFI prediction

**Fig. 2.**
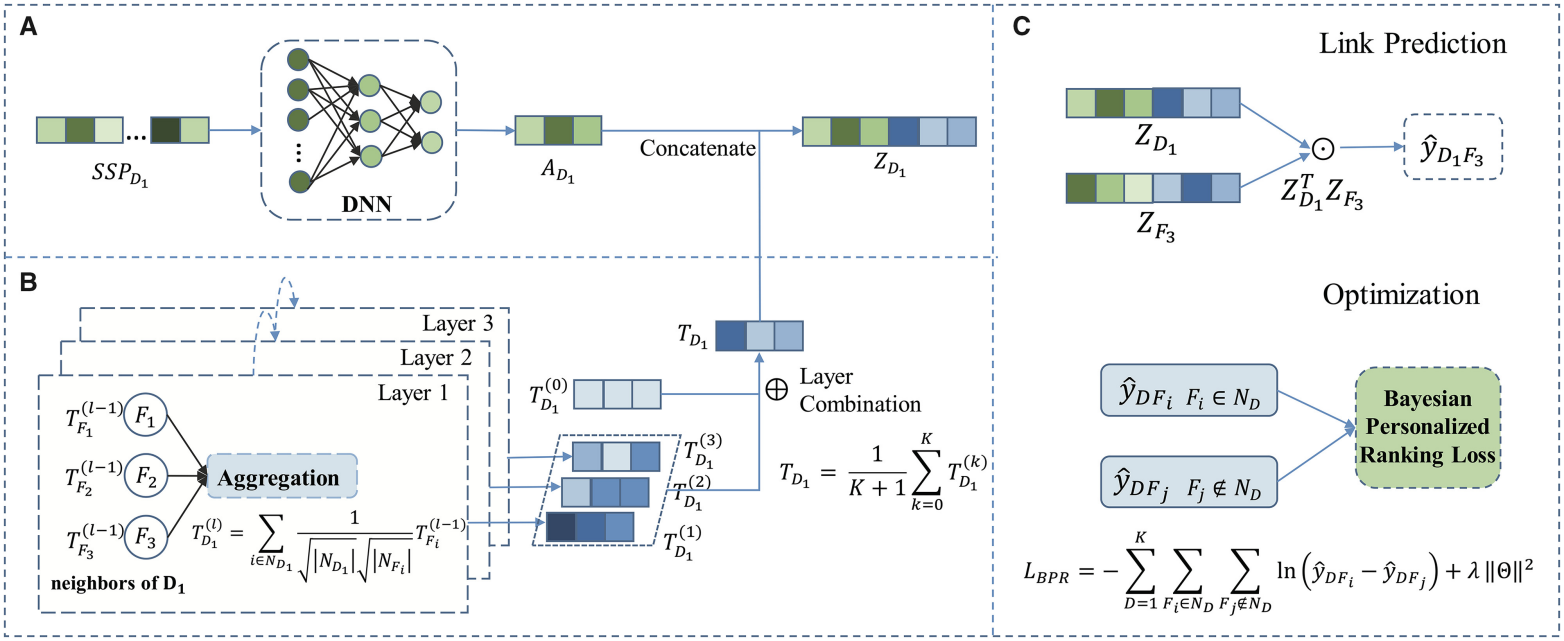
The framework of DFinder. We identify the interaction between *D*_1_ and *F*_3_ as an example to describe the process of our model. DFinder contains three main parts. (**A**)Attribute feature extraction. We use a three-layer DNN to learn the embedding of node $A_{D_{1}}$ in feature space. (**B**) Structure feature extraction. We use a simplified GCN-based method, which only contains the neighborhood aggregation operation to learn the embedding of node in topology space. For instance, in the first layer, drug *D*_1_ aggregates the feature of its neighbors (*F*_1_, *F*_2_ and *F*_3_) by simple weighted sum aggregator. Then, we combine the embeddings obtained at each layer to form the final topological feature $T_{D_{1}}$. (**C**) Finally, we concatenate the topological feature vector $T_{D_{1}}$ and attribute feature vector $A_{D_{1}}$ as the final representation $Z_{D_{1}}$. The attribute and structure features for food constituents are extracted in the same way as drug features. And the final representation of food constituent *F*_3_ is concatenated from its corresponding attribute and structure features. Finally, we use the inner product to get the score between *D*_1_ and *F*_3_ and use the BPR loss to optimize the model

The DFI network we obtain in Section 2.1 is a bipartite graph containing two types of nodes (drugs and food constituents) and one type of edges (interactions between drugs and food constituents). The overall framework of the DFinder is shown in Figure 2. The key idea of this model is to combine the node feature and topological structure to obtain a better representation of a drug (or food constituent). In topology space, we adopt a simplified GCN-based method to learn the topological structure of a network and obtain embedding *T_D_* of nodes. In feature space, a DNN is used to reduce the dimension of original node attribute information to learn embedding *A_D_* of the node. There are four layers in the DNN. The input layer has 2159 neurons. The first and second hidden layers have 1024 and 512 neurons, respectively. The output layer has 64 neurons. We use the ReLu function as the activation function in each layer. Then, we obtain the final embedding *Z_D_* of the node by concatenating *T_D_* and *A_D_*.

#### 2.2.1 Structure feature extraction

In this section, we describe how to extract structure features from the network. As a neural network designed to learn graph data, GCN has shown great popularity in solving graph analysis problems, such as node classification (Abu-El-Haija *et al.*, 2019, 2020), graph classification (Jiang *et al.*, 2020; Xie *et al.*, 2020), link prediction (Li *et al.*, 2019) and recommendation (Fan *et al.*, 2019). A typical GCN (Kipf and Welling, 2016a) layer consists of three operations: feature transformation, neighbor aggregation and non-linear activation. The propagation rule can be defined as:

(1) $$Z^{\left(l+1\right)}=\sigma \left({\tilde{D}}^{-\frac{1}{2}}\tilde{A}{\tilde{D}}^{-\frac{1}{2}}Z^{\left(l\right)}W^{l}\right),$$

where $\tilde{A}=A+I_{N}$ is the adjacency matrix with added self-connections. *I_N_* is the identity matrix. $\tilde{D}$ is the degree matrix, where ${\tilde{D}}_{ii}=\sum_{j}{\tilde{A}}_{ij}$; *W^l^* is a layer-specific trainable weight matrix; σ(·) denotes an activation function.

However, He *et al.* (2020) find that feature transformation and non-linear activation in standard GCN have no positive effect on recommendation performance. They propose LightGCN only containing the neighborhood aggregation operation, which is easier to be trained and more effective. The goal of the recommendation system is to mine the relationship between users and items, which is similar to the link prediction problem of biological networks. Inspired by their work, the proposed model only employs the simple weighted sum aggregator to learn the embeddings of drugs and food constituents. The propagation rule on the DFI network is defined as:

(2) $$T_{D}^{\left(l+1\right)}=\sum_{F\in N_{D}}\frac{1}{\sqrt{\left|N_{D}\right|}\sqrt{\left|N_{F}\right|}}T_{F}^{\left(l\right)},$$

(3) $$T_{F}^{\left(l+1\right)}=\sum_{D\in N_{F}}\frac{1}{\sqrt{\left|N_{F}\right|}\sqrt{\left|N_{D}\right|}}T_{D}^{\left(l\right)},$$

where *N_D_* and *N_F_*, respectively, denote the neighbor set of drug *D* and food constituent *F* in the DFI network. The symmetric normalization term $1/\sqrt{\left|N_{F}||N_{D}\right|}$ can avoid the scale of embeddings increasing with graph convolution operations. After the *K*-th graph convolution operation, we get the final representations of drugs (and food constituents) by combining the embeddings obtained at each layer:

(4) $$T_{D}=\frac{1}{K+1}\sum_{k=0}^{K}T_{D}^{\left(k\right)},$$

(5) $$T_{F}=\frac{1}{K+1}\sum_{k=0}^{K}T_{F}^{\left(k\right)}.$$

In this way, we can learn the node embeddings that capture the topology information *T_D_* for drug *D* and *T_F_* for food constituent *F* in the topology space.

#### 2.2.2 Attribute feature extraction

In the link prediction task, the original attribute information of the entity to be predicted may have a great impact on the performance of the prediction. To improve the effect of prediction, a lot of work studies how to use attribute information for entities. For example, DeepDDI (Ryu *et al.*, 2018) adopted a simplified molecular-input-line-entry system (SMILE) description to establish a structural similarity profile (SSP). The SSP is a feature vector that can capture structure features of individual drugs and food compounds. The SSP contains pairwise structural similarity scores obtained from the comparison with the 2159 approved drugs of DrugBank. In addition, DeepDDI compared the performance of DeepDDI using four different feature vectors: SSP, molecular descriptor profile and feature vectors generated by two state-of-the-art vectorization methods Molecular Autoencoder (Gómez-Bombarelli *et al.*, 2018) and Mol2vec (Jaeger *et al.*, 2018). SSP slightly outperforms the other three feature vectors when collaborating with the DeepDDI framework, which proves that SSP can effectively capture a unique structural feature of a given drug or food component. The specific calculation process of SSP is shown in Supplementary Figure S1. In the proposed model, we adopt SSP as the attribute information (feature vector) for drug and food constituent. First, we generate an SSP for each node as original attribute information. Then, we use a three-layer DNN as the feature extractor to learn the attribute feature from the original SSP. After inputting SSP into DNN, we finally get the low-dimensional embedding as a feature vector for each node in the feature space.

### 2.3 Feature fusion of different spaces

We concatenate structure features and attribute features to generate the final representation of each node:

(6) $$Z_{D}=\left(T_{D}||A_{D}\right);Z_{F}=\left(T_{F}||A_{F}\right),$$

where *Z_D_* and *Z_F_* denote the final embedding of drug and food constituent, respectively, *A_D_* and *A_F_* represent the attribute feature of the drug entity and food consistent entity, respectively and || denotes concatenation operation.

### 2.4 Model training

We introduce the prediction of DFIs and the optimization of our model in this section. The DFI prediction is defined as the inner product (Long *et al.*, 2020) of the drug and food constituent’s final representations. In detail, given a drug node *D_i_* and a food constituent node *F_j_*, $Z_{D_{i}}$ and $Z_{F_{j}}$ are their final representations, respectively. The probability that there is an interaction between *D_i_* and *F_j_* can be calculated as:

(7) $${\hat{y}}_{D_{i}F_{j}}=Z_{D_{i}}^{T}\cdot Z_{F_{j}},$$

where ${\hat{y}}_{D_{i}F_{j}}$ is the interaction score between drug node *D_i_* and food constituent node *F_j_*.

We used the BPR loss as the objective function for model optimization (Rendle *et al.*, 2012). The BPR loss is a pairwise personalized ranking loss, and has been widely used in recommendation systems. The training data of BPR consists of positive pairs (observed DFIs) and negative pairs (unobserved DFIs), and it encourages the prediction of observed interactions higher than unobserved interactions.

(8) $$L_{\mathrm{BPR}}=-\sum_{D=1}^{M}\sum_{F_{i}\in N_{D}}\sum_{F_{j}\notin N_{D}}ln\sigma \left({\tilde{y}}_{DF_{i}}-{\tilde{y}}_{DF_{j}}\right)+\lambda {\left\|\Theta \right\|}^{2},$$

where σ(·) is the sigmoid function. Θ is the embedding of the 0-th layer. $\Theta =\left[T_{D_{1}}^{\left(0\right)},\ldots ,T_{D_{M}}^{\left(0\right)},T_{F_{1}}^{\left(0\right)},\ldots ,T_{F_{N}}^{\left(0\right)}\right]$. *λ* is the parameter to control the *L*_2_ regularization strength. We use the mini-batch Adam (Kingma and Ba, 2014) as the optimizer, which can calculate the learning rate adaptively for each parameter.

## 3 Results

To evaluate the performance of DFinder, we test our model on DrugBank-DFI and PubMed-DFI, respectively. DrugBank-DFI is generated from DrugBank containing 1784 interactions. PubMed-DFI is generated by a co-occurrence-based text mining method from PubMed containing 13 580 interactions.

### 3.1 Experimental settings

#### 3.1.1 Data generation

The DFIs in DrugBank-DFI and PubMed-DFI are considered positive samples. We take all the unobserved DFIs in the two datasets as negative samples. For positive samples, we randomly select 20% of the whole observed DFIs as the testing set and use the remaining 80% as the training set. For negative samples, the training and testing sets are divided in the same way as positive samples.

#### 3.1.2 Performance evaluation

In this work, the identification of DFIs can be considered a link prediction task. Thus, we adopt area under the receiver operating characteristic curve (AUROC) and area under the precision-recall curve (AUPR) to evaluate our model. As far as we know, there are only a few computational methods designed for DFI identification, so we mainly compare our method with the graph embedding methods, which are developed for the link prediction task on the graph. In the evaluation test, we compare DFinder with other 12 algorithms, which contain 11 graph representation learning methods and preferential attachment (PA) method (Liben-Nowell and Kleinberg, 2003), where graph representation learning methods can be categorized into three groups: MF-based methods [Laplacian (Belkin and Niyogi, 2003), GF (Ahmed *et al.*, 2013), SVD, GraRep (Cao *et al.*, 2015) and HOPE (Ou *et al.*, 2016)], random walk-based methods [DeepWalk (Perozzi *et al.*, 2014), node2vec (Grover and Leskovec, 2016), struc2vec (Ribeiro *et al.*, 2017)] and neural network-based methods [LINE (Tang *et al.*, 2015), SDNE (Wang *et al.*, 2016) and GAE (Kipf and Welling, 2016b)]. The PA method estimates the probability of the co-occurrence of an edge (A, B) as a function of the product of degrees of end nodes A and B. To fair the comparison, in the performance evaluation of PA, we keep the node degree distribution similar in the positive edge set and negative edge set. During the performance evaluation, each dataset was randomly separated five times. Detailed information on these methods can be found in the Supplementary Material.

### 3.2 Performance evaluation on DrugBank-DFI

**Fig. 3.**
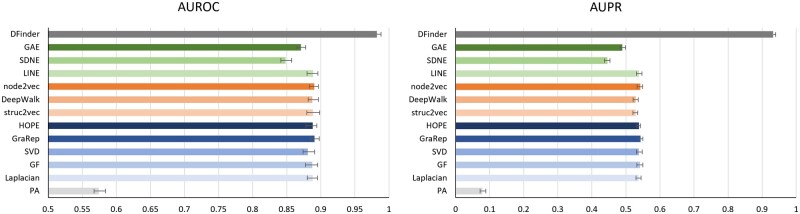
DFinder improves DFI prediction performance on DrugBank-DFI dataset. We compared the DFI prediction performance of DFinder to other approaches. In the figure, the left and right ones represent the AUROC and AUPR results of different methods, respectively

We first adopt the DrugBank-DFI dataset to validate the performance of DFinder. The error bar chart of AUROC and AUPR on the DrugBank-DFI dataset is shown in Figure 3. The detailed performance of AUROC and AUPR on the DrugBank-DFI dataset can be seen in Table 1. We compare DFinder with the 12 existing algorithms, we mentioned in Section 3.1.2. The experiment results demonstrate that DFinder has significantly improved the performance of DFI identification. In detail, the AUROC of DFinder is 9.17% higher than other methods (from 0.57 to 0.89), and AUPR is 38.96% higher than other methods (from 0.08 to 0.54). The reason for the performance improvement is that DFinder can not only aggregate the topological neighbors’ feature information in the DFI network but also introduce abundant attribute information for nodes. By fuzing node features and topological structures, DFinder can learn better representations for drugs and food constituents. In addition, our model learns the representations of nodes in an end-to-end way. In summary, DFinder outperforms the other 12 methods, which we use as baseline models in the identification of DFIs.

**Table 1.** The detailed AUROC and AUPR performance of DrugBank-DFI dataset

	Method	AUROC	AUPR	*F*1-score	Recall	Precision
MF-based	Laplacian	0.8882	0.5377	0.3212	0.2211	0.5874
	GF	0.8875	0.5419	0.377	0.2763	0.5932
	SVD	0.8810	0.5376	0.3811	0.2763	0.614
	GraRep	0.8906	0.5430	0.3943	0.2895	0.618
	HOPE	0.8881	0.5384	0.3922	0.2921	0.5967
Random walk-based	DeepWalk	0.8875	0.5283	0.3365	0.2368	0.5807
	node2vec	0.8905	0.5420	0.3308	0.2263	0.6143
	struc2vec	0.8884	0.5285	0.3949	0.2868	0.6337
Neural net-based	LINE	0.8888	0.5381	0.3709	0.2684	0.6
	SDNE	0.8483	0.4460	0.0001	0.0001	0.0001
	GAE	0.8709	0.4896	0.313	0.229	0.4943
	PA	0.5741	0.0788	0.1588	0.1592	0.1584
	DFinder	**0.9823**	**0.9326**	**0.8722**	**0.8733**	**0.8711**

*Note*: Bolded numbers are the best performance.

### 3.3 Performance evaluation on PubMed-DFI

**Fig. 4.**
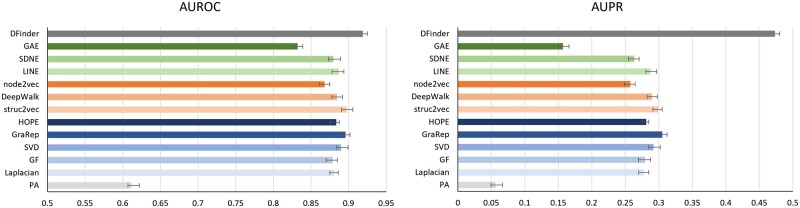
DFinder improves DFI prediction performance on PubMed-DFI dataset. We used the same baseline models as what we used on DrugBank-DFI. In the figure, the left one represents the AUROC results and the right one represents the AUPR results of different methods

To further explore the performance of our model, we also apply DFinder to PubMed-DFI to evaluate its performance. Compared with all baseline models, the proposed DFinder generally achieves the best performance according to AUROC and AUPR (Table 2). The error bar chart of AUROC and AUPR on PubMed-DFI dataset is shown in Figure 4. In detail, the AUROC of the DFinder is 2.13% higher than other methods (from 0.61 to 0.90), and AUPR is 16.83% higher than other methods (from 0.06 to 0.31). DFinder consistently outperforms all the graph representation learning methods, indicating the effectiveness of combining structure information of the network and attribute information of node as the representation of nodes. In addition, graph representation learning methods always follow a two steps pipeline: low-dimensional representations are first learned for nodes and then used as features for different downstream tasks. The experimental results also show that the end-to-end paradigm can learn a better representation for downstream tasks. In summary, this experiment demonstrates the effectiveness of the DFinder.

**Table 2.** The detailed AUROC and AUPR performance of PubMed-DFI dataset

	Method	AUROC	AUPR	*F*1-score	Recall	Precision
MF-based	Laplacian	0.8810	0.2779	0.5572	0.0984	0.5591
	GF	0.8790	0.2797	0.1336	0.0759	**0.6**
	SVD	0.8901	0.2921	0.1652	0.0981	0.5474
	GraRep	0.8959	0.3051	0.1768	0.1049	0.5619
	HOPE	0.8835	0.2811	0.1571	0.09	0.5993
Random walk-based	DeepWalk	0.8845	0.2896	0.1632	0.0961	0.5572
	node2vec	0.8681	0.2575	0.1427	0.0815	0.5729
	struc2vec	0.8977	0.2991	0.1755	0.1049	0.5356
Neural net-based	LINE	0.8868	0.2877	0.1606	0.0934	0.5738
	SDNE	0.8799	0.2634	0.0035	0.0018	0.375
	GAE	0.8322	0.1570	0.0024	0.0012	0.4444
	PA	0.6119	0.0567	0.1288	0.1293	0.1283
	DFinder	**0.9190**	**0.4734**	**0.5353**	**0.5354**	0.5353

*Note*: Bolded numbers are the best performance.

### 3.4 Performance comparison with DDI-prediction methods

Since the drug and food constituents have been represented as molecules, the methods designed for predicting DDIs are able to predict the DFI by treating the DFI network as a homogeneous DDI network. To further evaluate the performance of our method, we compare DFinder with seven state-of-the-art DDI-prediction methods, including CASTER (Huang *et al.*, 2020), MR-GNN (Xu *et al.*, 2019), GCN-BMP (Chen *et al.*, 2020), EPGCN-DS (Sun *et al.*, 2020b), SSI-DDI (Nyamabo *et al.*, 2021), DeepDrug (Yin *et al.*, 2022) and DeepDDI (Ryu *et al.*, 2018). Detailed information on these methods can be found in the Supplementary Material.

**Fig. 5.**
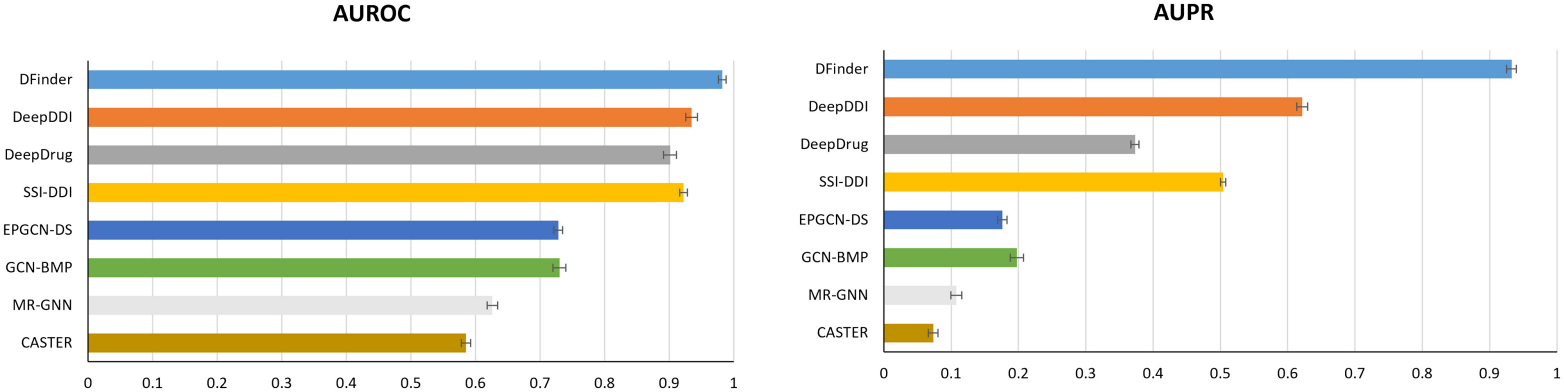
Performance comparisons between DFinder and seven DDI-prediction methods on the DrugBank-DFI dataset. In the figure, the left and right ones represent the AUROC and AUPR results of different methods, respectively

**Fig. 6.**
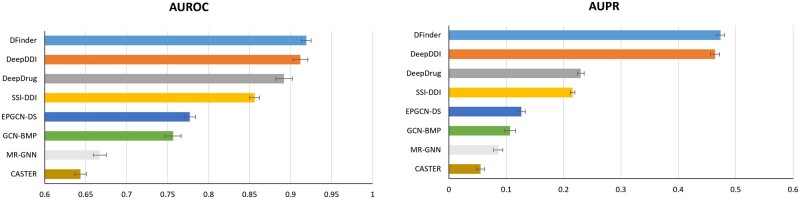
Performance comparisons between DFinder and seven DDI-prediction methods on the PubMed-DFI dataset. We used the same baseline models as what we used on DrugBank-DFI. In the figure, the left one represents the AUROC results and the right one represents the AUPR results of different methods

To fair the comparison, all these experiments are performed in the same way as DFinder. The compared methods use the DFI network and SMILE-based attribute features on nodes as inputs and are trained in the default settings. The detailed performance metrics, including AUROC, AUPR, *F*1-score, recall and precision, of eight methods on DrugBank-DFI and PubMed-DFI are shown in Table 3. The performance of AUROC and AUPR metrics on DrugBank-DFI is shown in Figure 5. DFinder performs the best in all of the metrics. For AUROC, DFinder reaches 0.98, which is 4.75% higher than other methods (ranging from 0.59 to 0.93). For AUPR, DFinder reaches 0.93, which is 31.1% higher than other methods (ranging from 0.07 to 0.62). And we illustrate the AUROC and AUPR metrics on PubMed-DFI in Figure 6. In detail, the AUROC of DFinder is the best (0.92), followed by DeepDDI (0.91) and DeepDrug (0.89). The AUPR of DFinder is also the best (0.47), followed by DeepDDI (0.46) and DeepDrug (0.23). From the above results, we can see our DFinder performs better than those DDI-prediction methods. These experiments indicate that those DDI-prediction methods treating the DFI network as a homogeneous graph may lose the specific heterogeneous topology features underlying DFI networks, and highlight the necessity of designing specific DFI prediction frameworks, like DFinder.

**Table 3.** Performance comparison between DFinder and seven DDI-prediction methods on DrugBank-DFI and PubMed-DFI datasets

Dataset	Method	AUROC	AUPR	*F*1-score	Recall	Precision
DrugBank-DFI	CASTER	0.5853	0.0733	0.0743	0.0741	0.0744
	MR-GNN	0.6262	0.1076	0.1673	0.167	0.1675
	GCN-BMP	0.7301	0.1977	0.2288	0.2285	0.2291
	EPGCN-DS	0.7283	0.176	0.0032	0.0016	0.1
	SSI-DDI	0.922	0.5041	0.4058	0.2992	0.6491
	DeepDrug	0.9013	0.3733	0.393	0.3923	0.3936
	DeepDDI	0.9348	0.6217	0.4162	0.2879	0.754
	DFinder	**0.9823**	**0.9326**	**0.8722**	**0.8733**	**0.8711**
PubMed-DFI	CASTER	0.6437	0.0551	0.1003	0.1002	0.1003
	MR-GNN	0.6672	0.086	0.1425	0.1425	0.1425
	GCN-BMP	0.7566	0.1065	0.1691	0.1691	0.1691
	EPGCN-DS	0.7769	0.1262	0.0229	0.0117	0.6234
	SSI-DDI	0.856	0.2156	0.0891	0.0485	**0.5575**
	DeepDrug	0.8919	0.2299	0.2975	0.2975	0.2976
	DeepDDI	0.9119	0.4639	0.4787	0.4786	0.4788
	DFinder	**0.919**	**0.4734**	**0.5353**	**0.5354**	0.5353

*Note*: Bolded numbers are the best performance.

### 3.5 Evaluation of DFinder discoveries

To evaluate the prediction results of DFinder, we collect two other DFI resources [Food Interactions with Drugs Evidence Ontology (FIDEO) (Bordea *et al.*, 2020) and POMELO (Hamon *et al.*, 2017)] and one DDI resource [DDI2013 (Herrero-Zazo *et al.*, 2013)]. The FIDEO is an ontology used for annotation and retrieval of scientific articles about food–drug interactions (Bordea *et al.*, 2020). We extract 1751 DFIs from the FIDEO after preprocessing. And after decomposing the food into food constituents, we obtained 10 938 DFIs. The POMELO is a Medline corpus with manually annotated food–drug interactions (Hamon *et al.*, 2017). We extract 2180 drug–food–constituent interactions from it after manually removing duplicated records, and records representing ‘no effect’ or containing unclear food or drug entities. The DDI corpus (named DDI2013 in this work) is an annotated corpus for DDIs. After removing the duplication, we obtained 4060 DDIs for downstream analysis. Note that, the POMELO-DFI and FIDEO-DFI are bipartite graphs, while DDI2013-DDI is a homogeneous graph in spite of some drug compounds being also food constituents. The detailed description of data sources and preprocessing can be found in the Supplementary Material.

**Fig. 7.**
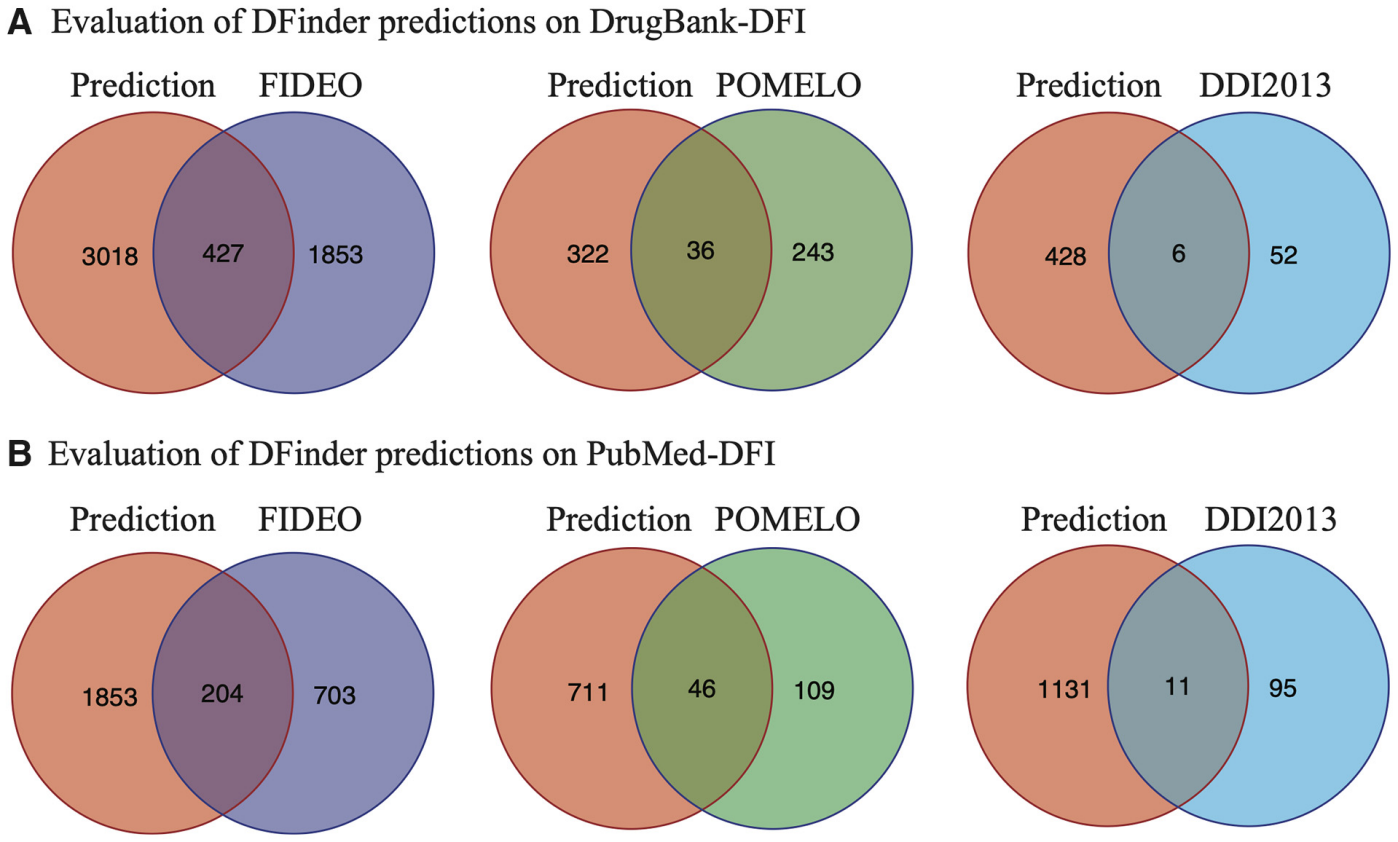
Evaluation of DFinder predictions on DrugBank-DFI and PubMed-DFI using FIDEO-DFI, POMELO-DFI and DDI2013 evaluation datasets. Comparisons were limited to the common node entities that are in the DFinder dataset and evaluation dataset. The predicted edges were prioritized by the top 20% of prediction probabilities. (**A**) Venn diagrams of DFinder predictions on DrugBank-DFI with three evaluation datasets. (**B**) Venn diagrams of DFinder predictions on PubMed-DFI with three evaluation datasets

We use the FIDEO-DFI, POMELO-DFI and DDI2013-DDI to evaluate the results of DFinder. The top 20% of DFI predictions, based on prediction probabilities, on DrugBank-DFI and PubMed-DFI are prioritized for evaluation. The comparisons are limited to the common node entities that are in our data and the evaluation data. Venn diagrams are used to illustrate the overlap between predicted DFIs and each evaluation data, as shown in Figure 7. In detail, in the FIDEO evaluation, we can see that 427 DFIs in FIDEO can be predicted by DFinder based on the DrugBank-DFI dataset, accounting for 18.7% of full FIDEO-DFIs that can be predicted. Two hundred and four (22.5%) DFIs in FIDEO can be predicted by DFinder based on the PubMed-DFI dataset. In the POMELO evaluation, 36 (12.9%) DFIs and 46 (29.7%) DFIs in the POMELO can be predicted by DFinder based on the DrugBank-DFI and PubMed-DFI, respectively. In the DDI2013 evaluation, we find only 6 and 11 DFIs in the DDI2013 predicted by the DFinder. This is mainly due to the small number of common node entities between DDI2013 (only drug entities) and our datasets (drug and food entities). We assume that there might be a large distinction between drug entities and food constituents. So we compare the full list of drugs from DrugBank and the full list of food constituents from FooDB, and we find there are only a small number of common entities between drugs and food constituents (Supplementary Fig. S2). This also demonstrates the importance of specifically building DFIs from multiple sources, which will compensate for each other.

We also test the performance of DFinder on the combined dataset based on the POMELO-DFI and the FIDEO-DFI. The performance evaluation process is the same as we have described in Section 3.1.2. The DFinder achieves an AUROC = 0.9802, AUPR = 0.8781 and *F*1-score = 0.8307, indicating stable performance on the combined dataset. To aid further validation, the DFinder-predicted DFIs, which can be replicated in at least one other resource, including the FIDEO-DFI, POMELO-DFI and DDI2013, have also been deposited on GitHub. There are 450 and 223 predicted DFIs in DrugBank and PubMed, respectively, that can be replicated with at least one of the other resources. For example, the DFI of ‘cyclosporine and sodium’ is not recorded in the DrugBank, but is predicted by DFinder. And this DFI is replicated in POMELO and FIDEO. Cyclosporine is used as an immunosuppressant medication, such as in patients with rheumatoid arthritis. Klawitter *et al.* have proven the genetic mechanism that a low-salt diet would induce higher sensitivity to cyclosporine (Klawitter *et al.*, 2012). The DFI of ‘fexofenadine and d-glucose’ is also predicted by DFinder. Fexofenadine is a medicine used in the treatment of allergy symptoms, such as hay fever and urticaria. The clinical has indicated that consuming large amounts of certain fruit juices may decrease the levels of fexofenadine in the body, while the d-glucose is enriched in the fruit juices. These evidences demonstrate the accuracy and practical value of our method.

## 4 Conclusion

In this article, we propose DFinder as a framework to identify interactions between drugs and food constituents by using a graph embedding-based method on DFI networks. Due to the lack of DFI data, we construct two DFI datasets named DrugBank-DFI and PubMed-DFI for link prediction tasks. We obtain the DrugBank-DFI dataset from DrugBank and get the PubMed-DFI dataset from PubMed by using the co-occurrence-based text mining method. We construct two DFI networks respectively based on the two datasets. DFinder combines node attribute features and topological structure features to learn the low-dimensional feature representations of drugs and food constituents. Then, we optimize the model in an end-to-end fashion. To evaluate the performance of our model, we compare DFinder with 11 graph representation learning methods and 7 DDI-prediction methods on these two networks. The experiment results show that the performance of DFinder is better than other methods on the DFI identification task.

## Funding

This work was supported by National Natural Science Foundation of China (No.62072376, 62102319).

*Conflict of Interest*: none declared.

## Supplementary Material

btac837_Supplementary_Data

## Data Availability

The data underlying this article are available in the article and in its online supplementary material.
