# Supplementary material for: DFinder: a novel end-to-end graph embedding-based method to identify drug–food interactions
Source: Bioinformatics. 2022 Dec 29;39(1):btac837. doi: 10.1093/bioinformatics/btac837 (PMC9828147; doi:10.1093/bioinformatics/btac837)
Supplement: btac837_Supplementary_Data [file btac837_supplementary_data.docx]

**Supplementary Information (SI Appendix)**

**DFinder: A novel end-to-end graph embedding-based method to identify drug-food interactions**

Tao Wang^1,2^, Jinjin Yang^1,2^, Yifu Xiao^1,2^, Jingru Wang^1,2^, Yuxian Wang^1,2^, Xi Zeng^1,2^, Yongtian Wang^1,2^ and Jiajie Peng^1, 2, *^

1School of Computer Science, Northwestern Polytechnical University, Xi’an, 710072, China

2Key Laboratory of Big Data Storage and Management, Northwestern Polytechnical University, Ministry of Industry and Information Technology, Xi’an, 710072, China

^*^To whom correspondence should be addressed. Email: [jiajiepeng@nwpu.edu.cn](mailto:jiajiepeng@nwpu.edu.cn)

In the supplementary document, we introduce five parts. In section 1, we introduce the details of the dataset. In section 2, we introduce how to calculate structural similarity profile (SSP). In section 3, we introduce the details of the baseline methods. In section 4, we introduce the evaluation of DFinder discoveries. In section 5, we talk about the potential bias in PubMed-DFI.

1. **Dataset**

To solve the lack of DFI resources, we construct two DFI datasets named DrugBank-DFI and PubMed-DFI. For DrugBank-DFI, we collect DFI related information directly from DrugBank database and then parse this information to generate the DrugBank-DFI dataset. For PubMed-DFI, we adopt the co-occurrence-based text mining method to obtain the PubMed-DFI dataset from PubMed. The detail information of these two DFI datasets is shown in Table 1.

1. **Calculation of structural similarity profile**

Structural similarity profile (SSP) contains pairwise structural similarity scores obtained from the comparison between the input drug and all the 2,159 approved drugs of DrugBank as a fixed comparison target. Structural similarity score was calculated by Tanimoto coefficient, which is defined as the number of common chemical fingerprints divided by the number of all the chemical fingerprints of the two drugs being compared. Chemical fingerprints of each drug were calculated by using extended-connectivity fingerprints of diameter 4 (ECFP4) with a Python package RDKit. The calculation process of SSP can be seen in Figure 1.

1. **The introduction of baseline methods**

In this paper, we compare twelve algorithms. They are matrix factorization-based methods (LEs[1], GF[2], SVD, GraRep[4] and HOPE[3]), random walk-based methods (DeepWalk[5], node2vec[6] and struc2vec[7]), neural network-based methods (LINE[8], SDNE[9] and GAE[10]), and Methods for DDI prediction, including DeepDDI[11], MR-GNN[12], CASTER[13], SSI-DDI[14], EPGCN-DS[15], DeepDrug[16], and GCN-BMP[17].

**3.1 Matrix factorization-based methods**

MF-based methods aim to factorize a data matrix into lower dimensional matrices while still keeping the manifold structure and topological properties hidden in the original data matrix. Laplacian Eigenmaps[1] and graph factorization[2] represent the relationships between nodes as graph adjacency matrix, Laplacian matrix or similarity matrix, and then adopt matrix factorization to obtain the embeddings. The difference between these methods is that they are based on different first-order matrices that capture the structure of graph, and they usually obtain a shallow embedding of nodes. In resent years, researchers focus on designing various high-order data proximity matrices to preserve the graph structure and propose various MF-based graph embedding learning methods. For example, GraRep[4] considers the high-order proximity of the network and designs k-step transition probability matrices for factorization. HOPE[3] also considers the high-order proximity. But different from GraRep, it adopts some well-known network similarity measures such as Katz Index and Common Neighbors to preserve network structures.

**3.2 random walk-based methods**

Random walk-based methods try to learn node representations by generating node sequences through random walks in graphs which generally have two steps. First, random walk is applied to generate node sequences in graph. Then the word2vec model is adopted to learn embeddings based on the generated sequences of nodes. In this way, the structural proximity of the graph can be preserved. DeepWalk[5] uses local information obtained from truncated random walks to learn latent representations by treating walks as the equivalent of sentences. Compared with DeepWalk, node2vec[6] mainly innovates in the way of random walk. Node2vec improves a flexible biased random walk, smoothly combining breadth-first sampling and depth-first sampling to obtain node sequences. Therefore, both local and global proximities are preserved. Struc2vec[7] is proposed for better modeling the structural identity (e.g. nodes in the

network may perform similar functions). Particularly, struct2vec first constructs a multi-layer weighted graph that encodes the structural similarity between nodes where each layer k is defined by using the k-hop neighborhoods of the nodes.

**3.3 Neural network-based methods**

LINE[8], the widely used embedding method can be regard as using a multilayer perceptron to approximate the first-order proximity and second-order proximity to learn node embedding. SDNE[9] adopts a deep autoencoder to preserve both global and local graph structure by modeling both first-order proximity (measured based on Laplacian Eigenmaps) and second-order proximity of nodes. GAE[10] utilizes a GCN encoder and an inner product decoder to learn node embeddings.

**3.4 Methods specifically designed for DDI prediction**

DeepDDI[11] was developed as a multi-label classification model that calculates structural similarity profiles (SSP) of DDIs. DeepDDI employs principal components analysis (PCA) to reduce the feature set size before feeding the rotated data into a feed-forward deep neural network (DNN). As an extended application, DeepDDI is also applied to drug-food constituent pairs to identify DFIs. Although DeepDDI can be used to identify DFIs, it is mainly designed for generating different types of DDIs.

MR-GNN[12] proposes a multi-resolution based architecture to extract node features from different neighborhoods of each node and it uses dual graph-state long short-term memory networks (LSTMs) to summarize local features of each graph and extracts the interaction features between pairwise graphs.

CASTER[13] develops a sequential pattern mining module rooted in the DDI mechanism to efficiently characterize functional sub-structures of drugs. CASTER uses an auto-encoding module that leverages both labeled and unlabelled chemical structure data to improve predictive accuracy and generalizability and a dictionary learning module that explains the prediction via a small set of coefficients that measure the relevance of each input sub-structures to the DDI outcome.

SSI-DDI (substructure-substructure interaction-drug-drug interaction)[14] operates directly on the raw molecular graph representations of drugs for richer feature extraction and breaks the DDI prediction task between two drugs down to identifying pairwise interactions between their respective substructures.

EPGCN-DS[15] is a DDI detection method that is based on molecular structures using graph convolutional networks and deep sets. EPGCN-DS proposed a more discriminative convolutional layer compared to conventional GCN and achieved permutation invariant prediction without losing the capability of capturing complicated interactions.

DeepDrug[16] uses graph convolutional networks to learn the graphical representations of drugs and proteins such as molecular fingerprints and residual structures to boost the prediction accuracy.

GCN-BMP[17] first uses RDKit to transform the pairwise SMILES strings into a pair of molecular graphs and then extract two types of structural information from molecular graphs. The two kinds of structural information are atom list and multi-channel adjacency matrix, which will be fed into the following Siamese GNN. Finally GCN-BMP adopts a HOLE-based neural network to compute the interaction probability of the input drugs.

1. **Evaluation of DFinder using POMELO, FIDEO, and DDI2013 datasets**

To evaluate the results of DFinder prediction, we collect two other DFI resources and one DDI resource.

POMELO is a medline corpus with manually annotated food-drug interactions [18]. We requested the current POMELO corpus from the authors (Hamon et al.) [18]. In the current POMELO corpus, there are 2,783 food entities and 4,953 drug entities recorded, however, only 853 food-drug relationships exist in the corpus. 449 food-drug relationships were discarded because the "relation_type" is "no_effect_on_drug" or the relationship did not have clear drug/food entities (such as drug/meal/food/breakfast etc.). Furthermore, 199 DFIs were filtered because of duplicated records. To be noted, we manually checked the duplication issue after consensus-based food entity renaming (such as renaming “grapefruit juice” as “grapefruit”). After the above processing, 205 drug-food interactions were left. We then decomposed the food into food constituents, as described in our Methods, to obtain 2,180 drug-food-constituent interactions (DFIs).

The Food Interactions with Drugs Evidence Ontology (FIDEO) [19] represents Food-Drug Interactions and underlying interaction mechanisms described in scientific publications, drug and adverse effects databases, and drug interactions compendia. The FIDEO [19] data was downloaded from <https://gitub.u-bordeaux.fr/erias/fideo>. We used the python package “rdflib” to extract the DFIs from the OWL raw file. 1,919 DFIs were extracted initially, and 168 records were filtered because of unclear entities or duplications, leaving 1,751 drug-food interactions. After decomposing the food into food constituents, we obtained 10,938 DFIs.

The last DDI corpus [20] (named DDI2013 in this work) is an annotated corpus for drug-drug interactions. The DDI2013 records were downloaded from https://github.com/isegura/DDICorpus. For the DDIs in DDI2013, there are also duplicated records. After removing the duplications, we obtained 4,060 DDIs for downstream analysis. Note that the POMELO-DFI and FIDEO-DFI are bipartite graphs, while DDI2013-DDI is a homogeneous graph in spite of some drug compounds being also food constituents.

The detail information of these three additional datasets is also shown in Table 1.

1. **Potential bias of co-occurrence-based PubMed-DFI**

The PubMed-DFI network was indeed a co-occurrence network. Like other co-occurrence networks, it can be affected by the not real interactions (https://en.wikipedia.org/wiki/Co-occurrence_network). We generated this network by searching the combinations of food constituents with drugs and calculated their co-occurrence frequencies in 3.3 million published papers in PubMed. The main hypothesis of this method is that if a biological entity appears in the same document as another biological entity, the two entities should have a high probability of being biologically related. This hypothesis has been used in several existing studies [21-25]. The co-occurrence-based DFIs have also been used in constructing the POMELO [18]. In the POMELO, the manually annotated corpus with food-drug interactions, 111 (54%) of the 205 food-drug interactions were annotated based on co-occurrence (with relation type = “relation”). To give an example, the food-drug interaction (clozapine-caffeine) shown in the Table 2 was annotated by POMELO based on the abstract description in the paper (PubmedID: 23104241) that “*The importance of caffeine consumption and Valproate comedication should be considered during clozapine dose adjustments to enhance its therapeutic response and safety profile*.” By semantic understanding, we can clearly infer that there is a candidate interaction between clozapine and caffeine. However, in the second example shown in Table 2, there seems less confidence in defining the food-drug interaction (hormone-caffeine) based on the description in the paper (PubmedID: 21721019) that “*Sulfotransferases (SULTs) are important phase II drug-metabolizing enzymes. Regulation of SULTs by hormones and other endogenous molecules is relatively well understood, while xenobiotic induction of SULTs is not well studied. Caffeine is one of the most widely consumed psychoactive substances. However, SULT regulation by caffeine has not been reported.*” In our method, to avoid the “errors” as much as possible, we applied more stringent criteria that we required more than or equal to 20 times co-occurring frequency together with two other rules (See Methods) to establish a drug-food association. The records in PubMed-DFI dataset actually are more like drug-food associations. But it is still valuable in developing and evaluating the robustness of our DFinder method since the co-occurrence-based FDIs largely expand the size of the drug-food bipartite network. Furthermore, the PubMed-DFI dataset could save tremendous efforts in further DFI validations by domain experts in the future.

We used a counting-based co-occurrence scoring approach together with other rules (See Method 2.1.2) to control the quality of PubMed-DFI dataset. There are 3 rules to control the quality: 1) keep food-drug pairs that co-occurred more than 20 times; 2) filter food-drug pairs with entities that are the same or similar; 3) filter pairs whose entity lacks the SMILE representation. The hard threshold of 20 times co-occurrence was defined based on the statistics of co-occurrence frequency, as shown in the Figure 3. Around 25% of “food-drug” pairs co-occurred more than 20 times. The counting-based co-occurrence scoring approaches can achieve high recall and require no training data or manually defined matching patterns [22]. Examples of using counting-based co-occurrence scoring include STRING [23] and DISEASES [24] as well as DisGeNet [25].

How to select a reasonable cut-off score is still an open question. We agree that some of the less-described pairs might also be true interactions. But we need the score to limit the number of interactions that have higher confidence and are more likely to be true positives. Setting the threshold lower will increase the coverage but also the false positives. In STRING [23], it is also required to set an arbitrary threshold to define the number of protein-protein interactions.

**SI Appendix Tables and Figures**

Table 1: Statistics of DrugBank-DFI, PubMed-DFI, POMELO-DFI, FIDEO-DFI，and DDI2013 datasets.

| Dataset Name | Drug # | Food constituent # | Interaction # | Density |
| --- | --- | --- | --- | --- |
| DrugBank-DFI | 143 | 213 | 1784 | 0.05857 |
| PubMed-DFI | 779 | 818 | 15890 | 0.02494 |
| POMELO-DFI^a^ | 123 | 212 | 2180 | 0.08360 |
| FIDEO-DFI^a^ | 600 | 309 | 11879 | 0.06407 |
| DDI2013 ^b^ | 1774 | NA | 4060 | 0.00258 ^b^ |

Notes: a. the entities in POMELO-DFI and FIDEO-DFI listed here contain those without SMILE representations. b. the density calculation of DDI2013 is different from other DFI datasets because DDI2013 is not a bipartite graph.

Table 2. Example of food-drug interactions in POMELO annotated based on entity co-occurrence.

| **paperID** | **drug_entity** | **food_entity** | **relation_type** |
| --- | --- | --- | --- |
| 23104241 | clozapine | caffeine | relation |
| 21721019 | hormone | caffeine | relation |


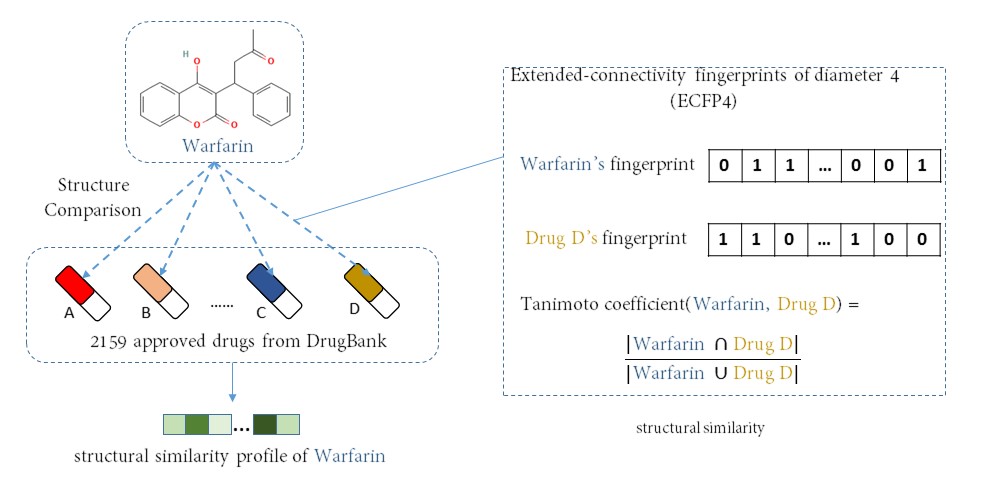


Figure 1. Calculation of structural similarity profile (SSP).

Figure 2. The intersection of food constituent entities in FooDB with drug entities in DrugBank.


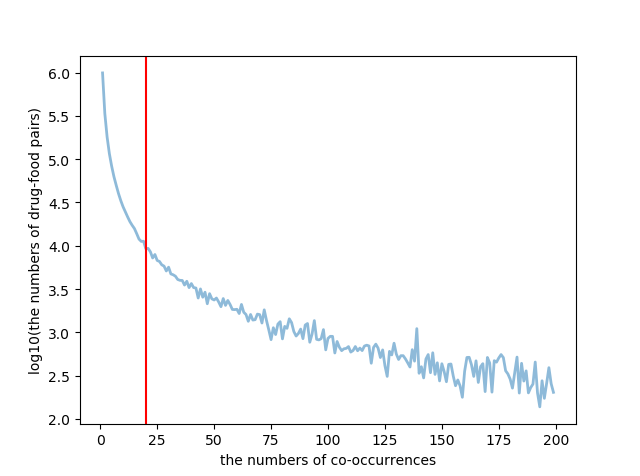


Figure 3. Frequency distribution of the number of “food-drug” co-occurrence in PubMed. The red vertical line indicates the threshold of 20 times of co-occurrences.

**Reference**

1. Belkin M, Niyogi P. Laplacian eigenmaps for dimensionality reduction and data representation[J]. Neural computation, 2003, 15(6): 1373-1396.
2. Ahmed A, Shervashidze N, Narayanamurthy S, et al. Distributed large-scale natural graph factorization[C]//Proceedings of the 22nd international conference on World Wide Web. 2013: 37-48.
3. Ou M, Cui P, Pei J, et al. Asymmetric transitivity preserving graph embedding[C]//Proceedings of the 22nd ACM SIGKDD international conference on Knowledge discovery and data mining. 2016: 1105-1114.
4. Cao S, Lu W, Xu Q. Grarep: Learning graph representations with global structural information[C]//Proceedings of the 24th ACM international on conference on information and knowledge management. 2015: 891-900.
5. Perozzi B, Al-Rfou R, Skiena S. Deepwalk: Online learning of social representations[C]//Proceedings of the 20th ACM SIGKDD international conference on Knowledge discovery and data mining. 2014: 701-710.
6. Grover A, Leskovec J. node2vec: Scalable feature learning for networks[C]//Proceedings of the 22nd ACM SIGKDD international conference on Knowledge discovery and data mining. 2016: 855-864.
7. Ribeiro L F R, Saverese P H P, Figueiredo D R. struc2vec: Learning node representations from structural identity[C]//Proceedings of the 23rd ACM SIGKDD international conference on knowledge discovery and data mining. 2017: 385-394.
8. Tang J, Qu M, Wang M, et al. Line: Large-scale information network embedding[C]//Proceedings of the 24th international conference on world wide web. 2015: 1067-1077.
9. Wang D, Cui P, Zhu W. Structural deep network embedding[C]//Proceedings of the 22nd ACM SIGKDD international conference on Knowledge discovery and data mining. 2016: 1225-1234.
10. Kipf T N, Welling M. Variational graph auto-encoders[J]. arXiv preprint arXiv:1611.07308, 2016.
11. Ryu J Y, Kim H U, Lee S Y. Deep learning improves prediction of drug–drug and drug–food interactions[J]. Proceedings of the National Academy of Sciences, 2018, 115(18): E4304-E4311.
12. Xu, N., Wang, P., Chen, L., Tao, J., & Zhao, J. (2019). Mr-gnn: Multi-resolution and dual graph neural network for predicting structured entity interactions. In Proceedings of the Twenty-Eighth International Joint Conference on Artificial Intelligence (IJCAI-19), pp. 3968-3974.
13. Huang, K., Xiao, C., Hoang, T., Glass, L., & Sun, J. (2020, April). Caster: Predicting drug interactions with chemical substructure representation. In Proceedings of the AAAI Conference on Artificial Intelligence (Vol. 34, No. 01, pp. 702-709).
14. Nyamabo, A. K., Yu, H., & Shi, J. Y. (2021). SSI–DDI: substructure–substructure interactions for drug–drug interaction prediction. Briefings in Bioinformatics, 22(6), bbab133.
15. Sun, M., Wang, F., Elemento, O., & Zhou, J. (2020, April). Structure-Based Drug-Drug Interaction Detection via Expressive Graph Convolutional Networks and Deep Sets (Student Abstract). In Proceedings of the AAAI Conference on Artificial Intelligence (Vol. 34, No. 10, pp. 13927-13928).
16. Yin, Q., Cao, X., Fan, R., Liu, Q., Jiang, R., & Zeng, W. (2022). DeepDrug: A general graph-based deep learning framework for drug-drug interactions and drug-target interactions prediction. biorxiv, 2020-11.
17. Chen, X., Liu, X., & Wu, J. (2020). GCN-BMP: Investigating graph representation learning for DDI prediction task. Methods, 179, 47-54.
18. Hamon, T., Tabanou, V., Mougin, F., Grabar, N., & Thiessard, F. (2017, September). POMELO: Medline corpus with manually annotated food-drug interactions. In Proceedings of the Biomedical NLP Workshop associated with RANLP 2017 (pp. 73-80).
19. Bordea, G., Nikiema, J., Griffier, R., Hamon, T., & Mougin, F. (2020, September). FIDEO: Food Interactions with Drugs Evidence Ontology. In 11th International Conference on Biomedical Ontologies.
20. Herrero-Zazo, M., Segura-Bedmar, I., Martínez, P., & Declerck, T. (2013). The DDI corpus: An annotated corpus with pharmacological substances and drug–drug interactions. Journal of biomedical informatics, 46(5), 914-920.
21. Fleuren, Wilco WM, and Wynand Alkema. "Application of text mining in the biomedical domain." Methods 74 (2015): 97-106.
22. Junge, A., & Jensen, L. J. (2020). CoCoScore: context-aware co-occurrence scoring for text mining applications using distant supervision. Bioinformatics, 36(1), 264-271.
23. Szklarczyk, D., Gable, A. L., Lyon, D., Junge, A., Wyder, S., Huerta-Cepas, J., ... & Mering, C. V. (2019). STRING v11: protein–protein association networks with increased coverage, supporting functional discovery in genome-wide experimental datasets. Nucleic acids research, 47(D1), D607-D613.
24. Pletscher-Frankild, S., Pallejà, A., Tsafou, K., Binder, J. X., & Jensen, L. J. (2015). DISEASES: Text mining and data integration of disease–gene associations. Methods, 74, 83-89.
25. Piñero, J., Bravo, À., Queralt-Rosinach, N., Gutiérrez-Sacristán, A., Deu-Pons, J., Centeno, E., ... & Furlong, L. I. (2016). DisGeNET: a comprehensive platform integrating information on human disease-associated genes and variants. Nucleic acids research, gkw943.
